# Microstructural and Metabolic Changes in Normal Aging Human Brain Studied with Combined Whole-Brain MR Spectroscopic Imaging and Quantitative MR Imaging

**DOI:** 10.1007/s00062-023-01300-3

**Published:** 2023-06-19

**Authors:** N. Mahmoudi, M. Dadak, P. Bronzlik, A. A. Maudsley, S. Sheriff, H. Lanfermann, X.-Q. Ding

**Affiliations:** 1 Institute of Diagnostic and Interventional Neuroradiology, Hannover Medical School, Hannover, Germany; 2 Department of Diagnostic and Interventional Radiology and Neuroradiology, St. Vincenz Hospital Paderborn, Paderborn, Germany; 3 Department of Radiology, University of Miami School of Medicine, Miami, FL USA

**Keywords:** Spin-echo planar spectroscopic imaging, Metabolic imaging and data analysis system, qMRI, Aging, Human brain metabolites

## Abstract

**Purpose:**

This study aimed to detect age-related brain metabolic and microstructural changes in healthy human brains by the use of whole-brain proton magnetic resonance spectroscopic imaging (^1^H‑MRSI) and quantitative MR imaging (qMRI).

**Methods:**

In this study, 60 healthy participants with evenly distributed ages (between 21 and 69 years) and sex underwent MRI examinations at 3T including whole-brain ^1^H‑MRSI. The concentrations of the metabolites N‑acetylaspartate (NAA), choline-containing compounds (Cho), total creatine and phosphocreatine (tCr), glutamine and glutamate (Glx), and myo-inositol (mI), as well as the brain relaxation times T2, T2’ and T1 were measured in 12 regions of interest (ROI) in each hemisphere. Correlations between measured parameters and age were estimated with linear regression analysis and Pearsonʼs correlation test.

**Results:**

Significant age-related changes of brain regional metabolite concentrations and tissue relaxation times were found: NAA decreased in eight of twelve ROIs, Cho increased in three ROIs, tCr in four ROIs, and mI in three ROIs. Glx displayed a significant decrease in one ROI and an increase in another ROI. T1 increased in four ROIs and T2 in one ROI, while T2’ decreased in two ROIs. A negative correlation of tCr concentrations with T2’ relaxation time was found in one ROI as well as the positive correlations of age-related T1 relaxation time with concentrations of tCr, mI, Glx and Cho in another ROI.

**Conclusion:**

Normal aging in human brain is associated with coexistent brain regional metabolic alterations and microstructural changes, which may be related to age-related decline in cognitive, affective and psychomotor domains of life in the older population.

**Supplementary Information:**

The online version of this article (10.1007/s00062-023-01300-3) contains supplementary material, which is available to authorized users.

## Introduction

The human brain governs our behavior and physical system through different ways, such as neuroendocrine, autonomic, immune and metabolic systems. Understanding of normal age effects on brain structures and brain metabolism may help to improve the quality of life in the older population and to recognize neurodegenerative alterations in patients prior to onset of symptoms. Proton magnetic resonance spectroscopy (^1^H‑MRS) and quantitative magnetic resonance imaging (qMRI) methods are important tools for investigation of age-related microstructural alterations that are invisible in conventional standard MR images. For example, with qMRI methods, brain maps of different tissue parameters, e.g., longitudinal (T1) or transverse (T2) relaxation times, can be derived, allowing quantitative measurements of the parameters from multiple brain structures. The changes of the parameter values reflect brain microstructural alterations [1–3]. Additionally, ^1^H‑MRS enables in vivo measurements of brain metabolites, including N‑acetylaspartate (NAA), which mirrors neuronal integrity, choline-containing compounds (tCho) mirroring cell membrane turnover, creatine and phosphocreatine (tCr) reflecting energy metabolism, myo-inositol (mI) reflecting gliosis and glutamine (Gln) and glutamate (Glu) or the combined signal from both (Glx), which function as excitatory neurotransmitters [4, 5]. ^1^H‑MRS has been used in numerous studies on aging human brain to estimate related brain metabolic changes; however, most of these studies have been carried out on one or a few small brain regions due to technical limitations in the spatial coverage of standard MRS acquisition techniques [6, 7], thus the provided information may not reflect the metabolic status of the whole brain. These technical limitations also hamper a combined use of ^1^H‑MRS and qMRI to estimate metabolic and microstructural changes of desired brain structures simultaneously, although linked knowledge about metabolic and microstructural changes may broaden our insights into aging processes in the human brain. This shortage has been overcome by a recently established whole-brain ^1^H‑MRS imaging technique (wbMRSI), which enables the measurement of brain regional metabolite concentrations from multiple brain structures with a single MR scan [8–10]. It has been used, for example, to simultaneously determine age-related metabolic changes over brain lobar structures or in multiple desired small regions of interest [11–15]. Eylers et al. demonstrated the feasibility of simultaneously studying the metabolic and microstructural changes of the aging human brain by combining long echo time wbMRSI and qMRI methods [16]; however, such studies are rare and more data are needed to gain knowledge and validate the findings. Therefore, the aim of this study was to evaluate microstructural and metabolic aging effects in healthy aging human brain by the use of a short echo time wbMRSI in combination with qMRI.

## Methods

### Subjects

For this study, 72 healthy subjects were recruited from the local population. The enrolled subjects had no systemic diseases such as neurological or psychiatric disorders, untreated chronic hypertension, and diabetes mellitus, or a history of brain trauma. To exclude those with potential cognitive or psychiatric impairments, each subject underwent two screening tests prior to the MR examination, the DemTect test with a score of 13 or above [17] and the Beck Depression Inventory II (BDI-II) with a score below 9 [18] as the expected range for healthy subjects. Subjects with abnormal results of the aforementioned screening tests (*n* = 5), incomplete MR examinations (*n* = 1), underweight (body mass index [BMI] ≤ 18, *n* = 1), obesity (body mass index ≥ 30, *n* = 3) or brain morphological abnormalities (*n* = 2) were excluded. Finally, 60 participants aged between 21 and 69 years (mean age = 45 years ± 15 years, 6 men and 6 women per age decade, mean BMI score = 24.3 ± 2.7, mean BDI-II score = 2 ± 2, and mean DemTect score = 17 ± 1) were included. The study was approved by the local ethics committee and conducted according to the principles expressed in the Declaration of Helsinki. Written consent was obtained from all subjects before the examinations.

### MR Examination and Data Processing

All subjects underwent a MR examination at 3T (Verio, Siemens, Erlangen, Germany) with a 12 channel phased array head coil [9, 10, 16]: For quantitative MR measurements the following scan sequences with a field of view of 256 × 208 mm^2^ and a 3 mm thickness in axial section were used: a T2-weighted turbo spin-echo sequence with 3 echoes (triple echo) (T2wTSE, TR/TE = 6640/8.7/70/131 ms, 150° flip angle), a T2*-weighted gradient-echo sequence with triple TE (T2*wGRE, TR/TE = 1410/6.42/18.42/30.42 ms, 20°flip angle), and a T1 weighted three-dimensional (3D) GRE sequence with two flip angles (T1wGRE, TR/TE = 15/1.64 ms, flip angles 5° and 25°). For wbMRSI a T1-weighted 3D magnetization prepared rapid gradient echo (MPRAGE) acquisition at 1‑mm isotropic resolution for anatomic reference and a volumetric spin-echo planar spectroscopic imaging (EPSI) sequence (TR/TE = 1550/17.6 ms, 50 × 50 voxels in-plane and 18 slices, over a field of view of 280 × 280 × 180 mm^3^) were used, where the EPSI acquisition included a second dataset obtained without water suppression that was used for several processing functions and for internal signal reference for the normalization of metabolite concentrations as described previously [19]. To ensure identifying the same anatomic structures, all scans were obtained with the same angulation. The T2 and T1 weighted images were inspected by two experienced neuroradiologists to exclude subjects with morphological abnormalities. Brain maps of the tissue parameters were derived from scanned MRI data. For quantifying transverse relaxation process of brain tissue, the parameters T2 (irreversible relaxation time), describing proton spin-spin interactions, and T2’ (reversible relaxation time), characterizing local magnetic field inhomogeneity, were considered. The T2*-maps (effective relaxation time) related to both mechanisms together were used to obtain T2’ according to the relationship 1/T2’ = 1/T2* − 1/T2. Brain T2-maps were obtained on the MR system with an extended image reconstruction using monoexponential fitting to the signal intensity decay curves of the data acquired with triple echo T2wTSE sequence. The T2*-maps were reconstructed in a similar way from the data acquired with triple echo T2*wGRE sequence, and the T1-maps from the data acquired with T1wGREsequence. Subsequently the T2’-maps were derived according to the relationship mentioned above.

Brain maps of metabolic parameters were derived from the wbMRSI data. Metabolite image reconstruction was made by analyzing the EPSI data in combination with MPRAGE data by use of the software package Metabolic Imaging and Data Analysis System (MIDAS), as previously described [8, 9]. Finally, the maps of the metabolites NAA, tCr, tCho, Glx, mI, and the accompanied spectral linewidth (sLW), as well as maps of relative cerebrospinal fluid (CSF) component were derived. [16, 19, 20].

### Region of Interest Analysis

**Fig. 1:**
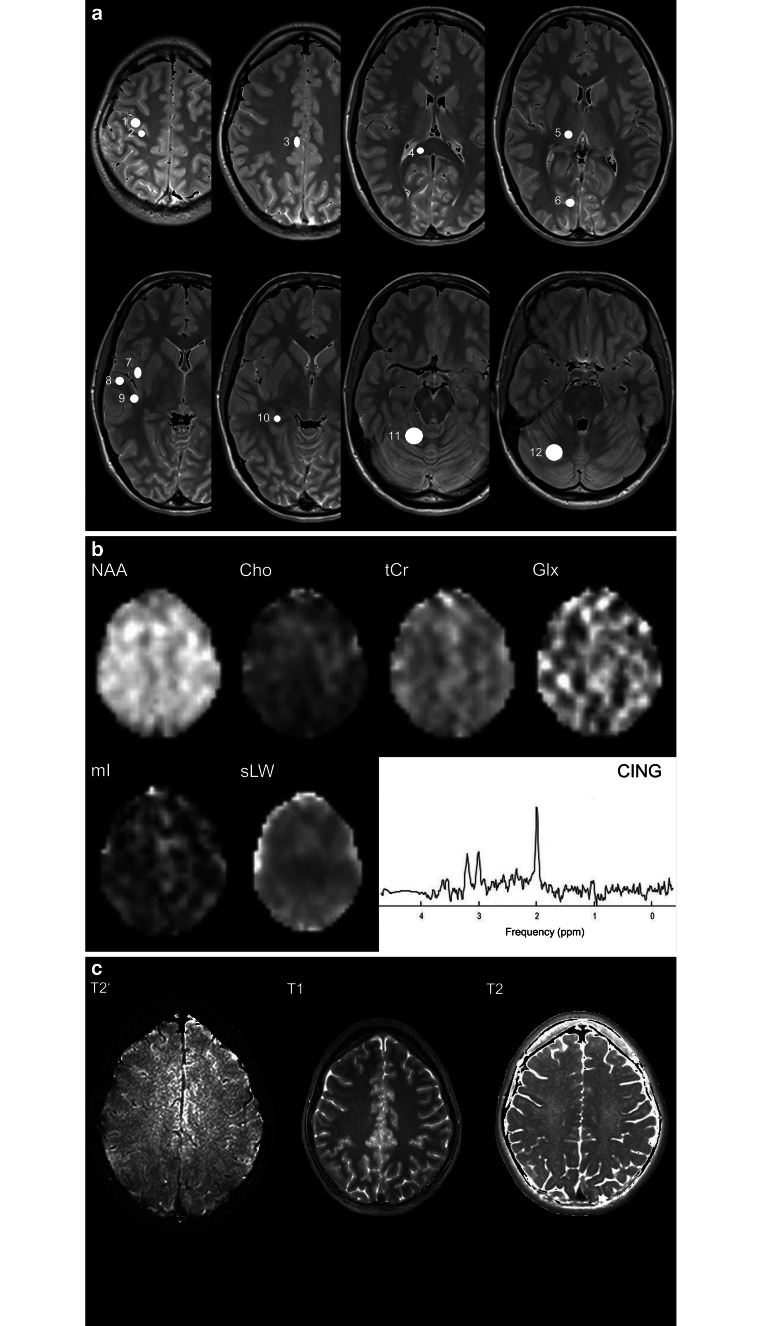
**a** Locations of each selected region of interest in the right brain hemisphere displayed as white filled circles on T2-weighted images of a 21-year-old healthy volunteer. The numbering represents the ROIs in the hand motor cortical area (1), postcentral gyrus (2), cingulate gyrus (3), splenium of the corpus callosum (4), thalamus (5), occipital area (6), temporal lobe lateral (7), temporal lobe medial (8), insular gyrus (9), hippocampus (10), cerebellum anterior lobe (11) and cerebellum posterior lobe (12). **b** Example metabolite maps of NAA, Cho, tCr, Glx, mI and spectral linewidth at the section level of the posterior cingulate gyrus with an example MR spectrum derived from wbMRSI. **c** Example tissue parameter maps of T2’, T1 and T2 at the section level of the posterior cingulate gyrus

The derived brain maps were used for region of interest analysis. The values of each parameter were estimated by using mean values over each of twelve regions of interest (ROIs) selected within each hemisphere, i.e., in hand motor cortical area (HCA), postcentral gyrus (PCEN), posterior cingulate gyrus (CING), splenium of the corpus callosum (SCC), thalamus (THAL), occipital area (OC), lateral temporal lobe (TLAT), medial temporal lobe (TMED), insular gyrus (INS), hippocampus (HIP), cerebellar anterior lobe (CANT) and cerebellar posterior lobe (CPOST), as shown in Fig. 1. All ROIs were identified on T1-weighted images and T2-weighted images, and carefully drawn as a circle or oval with an area between 21 mm^2^ (HIP) and 144 mm^2^ (CANT). Subsequently, the values of all parameters were obtained from each of the ROIs, where the values of T1, T2, and T2’ were determined in unit of ms and the values of metabolite concentrations, denoted as [NAA], [tCr], [tCho], [Glx] and [mI], were determined as ratios to tissue water and presented in institutional unit (i.u.). Additionally, the metabolite concentrations of all ROIs were corrected for CSF volume contribution according to Met’ = Met/(1 − f_csf_) for 0 < f_csf_ < 30%, where Met is the measured metabolite value, and f_csf_ is the fraction of CSF volume within the ROI measured from CSF maps [9].

### Statistical Analysis

All measured data were controlled according to the following quality criteria: values of those ROIs measured from metabolite maps with an accompanied linewidth larger than 12 Hz or measured from tissue parameter maps with a coefficient of variation (COV = standard deviation/mean value) higher than 25% were excluded from further analysis. Additionally, concerning the limitations of the sample size related to left handedness (*n* = 55 for right hand vs. 5 for left hand), we averaged the measured values of the corresponding ROIs in the left and right hemispheres.

The two-sided t‑test was used to estimate gender differences of the measured parameter values with a Bonferroni corrected significance level α = 0.05/12 = 0.004. Data from the ROIs showing significant gender differences were then analyzed separately for males and females in a further analysis. The two-sided t‑test revealed significant differences between female and male values only for spectral linewidth in 2 ROIs (HCA and PCEN) and for T2’ in one ROI (INS). The correlation analyses of these data, performed using both male and female values separately and combined male and female values, did not reveal significant correlation with age. Therefore, the results derived from the combined male and female values are reported.

Pearson’s correlation test was used to estimate possible correlations of the measured values with age at each ROI. If a significant correlation was found, a linear regression analysis was carried out to estimate the age dependence. If both metabolic and tissue parameters showed age-related changes in a brain structure, Pearson’s correlation test was used to test for possible correlations between the metabolic and microstructural parameters. With the aim to explore brain areas that are most sensitive to aging effects and to reveal correlated metabolic and tissue parametric alterations with age, an uncorrected significance threshold of 0.05 was used for the analyses, with the results being reported together with the individual *p*-values.

Before statistical analysis, the distribution of the measured values was checked by using Kolmogorov-Smirnov test and Q‑Q plots. For non-normal distributed values, additional data analysis with a non-parametric Mann-Whitney U‑test was used to estimate gender differences and Spearman’s correlation tests were used to estimate the correlation to age. The results did not differ significantly from those obtained with parametric methods, therefore, the results derived from the parametric methods are reported in the following for all measured data.

The statistical analyses were performed with SPSS, Version 27 (SPSS IBM, Armonk, NY, USA).

## Results

Figure 1 shows the locations of selected regions of interest (ROI) in the right brain hemisphere, displayed as white filled circles or oval on the T2-weighted images of a 21-year-old female volunteer (Fig. 1a), the example tissue parameter maps and the metabolite maps at the section level of selected posterior cingulate gyrus ROI (Fig. 1b, c).

Metabolite concentrations [NAA], [tCr], [tCho], [Glx], and [mI], together with accompanied sLW measured from each ROI, as well as the values of T1, T2, and T2’, are drawn as scatter points against age groups in Supplemental Figs. 1 and 2. The corresponding decade mean values of each ROI, derived by averaging measured values of the healthy subjects within each age decade, are shown in Supplemental Table 1. The results of Pearson’s correlation test for correlations of the measured parameters with age are given in Table 1, and the results of the linear regression analysis are given in Table 2, with corresponding linear fits shown as diagram in Fig. 2a–c.

**Fig. 2:**
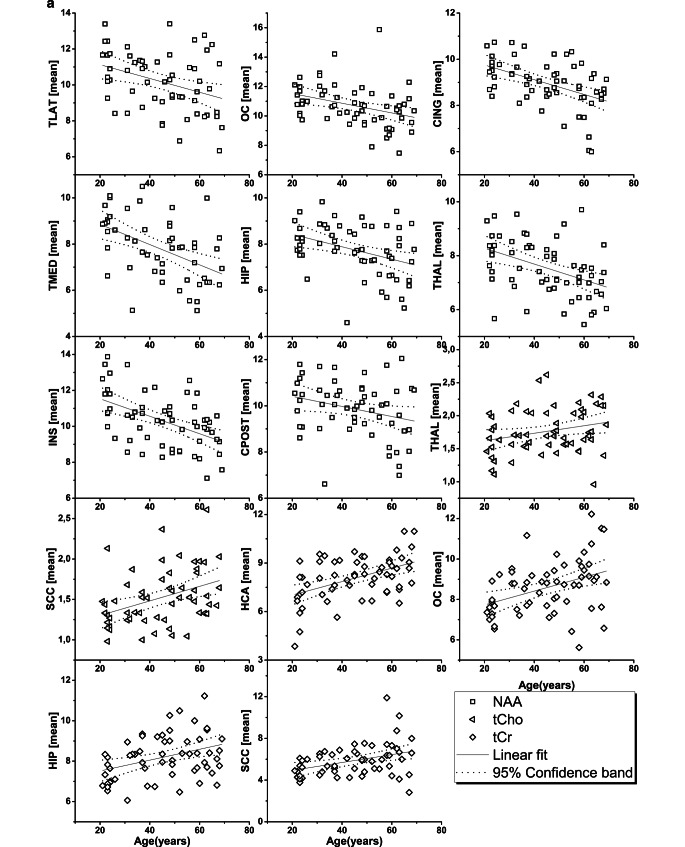
**a** Regional concentrations of NAA, tCho and tCr of regions of interest with age-related correlation. Metabolite concentrations are plotted against age and linear analysis was performed, showing linear fit and 95% confidence band. All regional metabolite concentrations were measured in ratio to internal water, and present in institutional units (i.u.). ROI: hand motor cortical area (*HCA*), posterior cingulate gyrus (*CING*), splenium of the corpus callosum (*SCC*), thalamus (*THAL*), occipital area (*OC*), lateral temporal lobe (*TLAT*), medial temporal lobe (*TMED*), insular gyrus (*INS*), hippocampus (*HIP*) and cerebellar posterior lobe (*CPOST*)

**Fig. 2:**
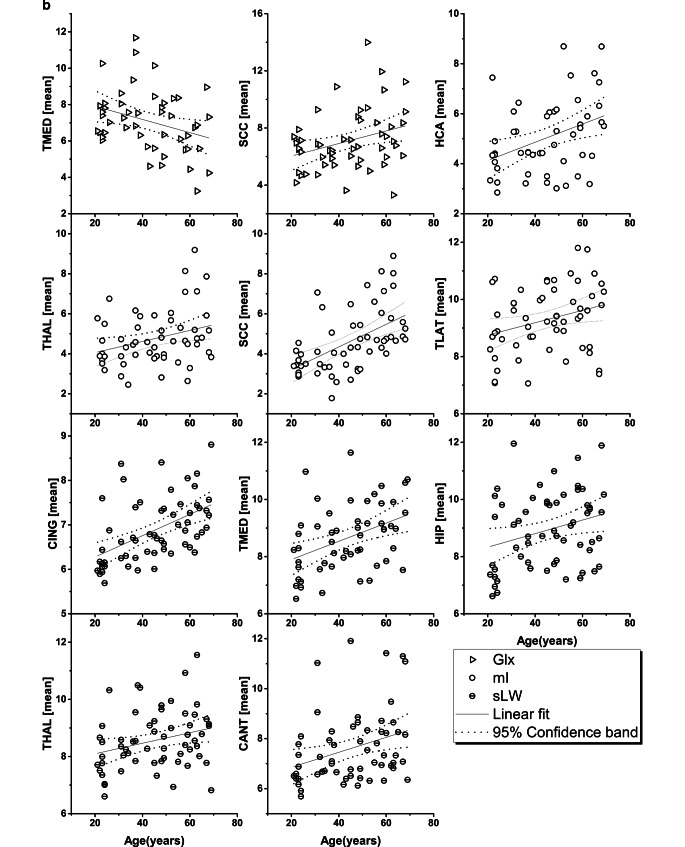
**b** Regional concentrations of Glx, mI and sLW of regions of interest with age-related correlation. Metabolite concentrations and spectral linewidths are plotted against age and linear analysis was performed, showing linear fit and 95% confidence band. All regional metabolite concentrations are presented in institutional units (i.u.) and sLW in hertz (Hz). ROI: hand motor cortical area (*HCA*), posterior cingulate gyrus (*CING*), splenium of the corpus callosum (*SCC*), thalamus (*THAL*), lateral temporal lobe (*TLAT*), medial temporal lobe (*TMED*), hippocampus (*HIP*) and cerebellar anterior lobe (*CANT*)

**Fig. 2:**
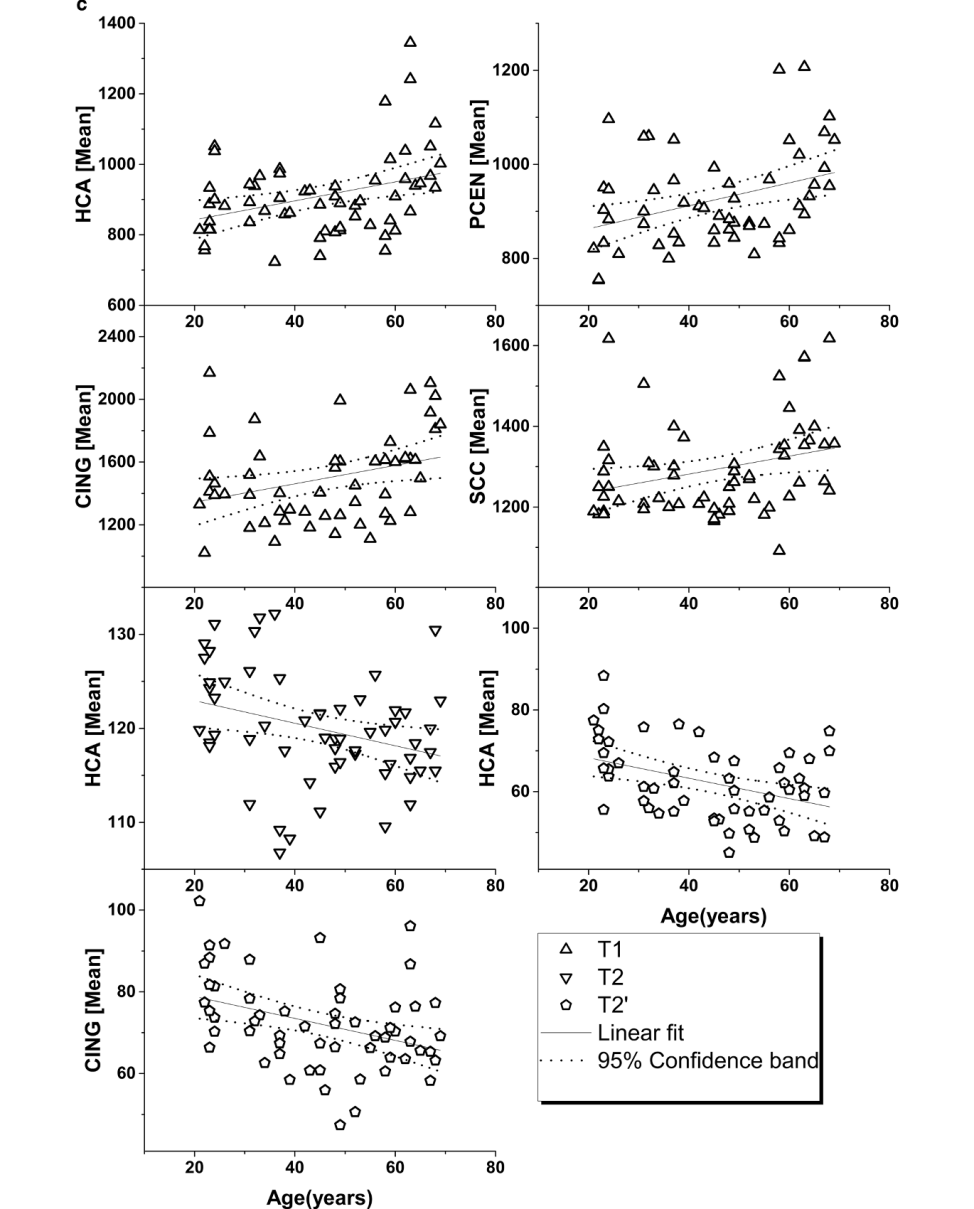
**c** Regional T1, T2 relaxation times (ms) of regions of interest with age-related correlation. Parameters are plotted against age and linear analysis was performed, showing linear fit and 95% confidence band. ROI: hand motor cortical area (*HCA*), postcentral gyrus (*PCEN*), posterior cingulate gyrus (*CING*) and splenium of the corpus callosum (*SCC*)

**Table 1:** Results of Pearson’s correlation test of the measured parameters with age

***ROI***	***NAA***			***tCho***			***tCr***			***Glx***			***mI***		
	*R*	*p*	*N*	*R*	*p*	*N*	*R*	*p*	*N*	*R*	*p*	*N*	*R*	*p*	*N*
HCA	0.100	0.453	59	0.241	0.066	59	**0.472**	**0.000**	**59**	0.082	0.550	55	**0.376**	**0.007**	**50**
PCEN	−0.058	0.659	60	0.199	0.128	60	0.244	0.060	60	−0.137	0.306	58	0.144	0.284	57
TLAT	**−0.355**	**0.006**	**58**	−0.130	0.345	55	0.036	0.786	58	−0.082	0.543	58	0.169	0.217	55
OCC	**−0.355**	**0.005**	**60**	0.078	0.558	59	**0.390**	**0.002**	**60**	−0.065	0.624	59	0.224	0.085	60
CING	**−0.474**	**0.000**	**60**	0.099	0.450	60	0.042	0.748	60	−0.149	0.266	58	0.234	0.085	55
TMED	**−0.485**	**0.000**	**53**	0.095	0.507	51	−0.224	0.111	52	**−0.317**	**0.027**	**49**	0.020	0.890	51
HIP	**−0.370**	**0.004**	**59**	0.012	0.929	59	**0.369**	**0.004**	**59**	−0.127	0.351	56	−0.050	0.710	58
THAL	**−0.432**	**0.001**	**60**	**0.255**	**0.049**	**60**	0.122	0.353	60	0.017	0.901	59	**0.302**	**0.025**	**55**
INS	**−0.494**	**0.000**	**60**	0.167	0.201	60	0.018	0.892	60	−0.060	0.650	59	0.201	0.130	58
CANT	−0.035	0.793	59	0.081	0.540	59	−0.095	0.472	59	−0.048	0.735	52	−0.023	0.869	56
CPOST	**−0.268**	**0.046**	**56**	−0.065	0.630	58	0.088	0.512	58	−0.125	0.378	52	0.105	0.437	57
SCC	0.054	0.687	58	**0.382**	**0.003**	**57**	**0.368**	**0.005**	**58**	**0.319**	**0.019**	**54**	**0.536**	**0.000**	**57**
***ROI***	***T1***			***T2***			***T2’***			***sLW***					
	*R*	*p*	*N*	*R*	*p*	*N*	*R*	*p*	*N*	*R*	*p*	*N*			
HCA	**0.353**	**0.006**	**60**	**−0.309**	**0.018**	**58**	**−0.407**	**0.002**	**54**	0.215	0.102	59			
PCEN	**0.372**	**0.004**	**57**	0.007	0.956	59	−0.250	0.070	53	0.206	0.114	60			
TLAT	0.171	0.195	59	−0.202	0.122	60	0.036	0.798	53	**0.286**	**0.030**	**58**			
OCC	0.030	0.825	57	−0.081	0.587	47	−0.174	0.200	56	0.155	0.237	60			
CING	**0.317**	**0.021**	**53**	0.124	0.344	60	**−0.370**	**0.005**	**57**	**0.509**	**0.000**	**59**			
TMED	−0.036	0.791	58	0.002	0.991	60	−0.045	0.768	46	**0.408**	**0.002**	**53**			
HIP	0.036	0.789	59	−0.100	0.445	60	−0.197	0.141	57	**0.276**	**0.034**	**59**			
THAL	0.122	0.354	60	0.085	0.519	60	−0.041	0.764	57	**0.273**	**0.035**	**60**			
INS	0.058	0.660	59	−0.131	0.356	52	−0.280	0.065	44	0.006	0.965	60			
CANT	−0.001	0.997	58	−0.233	0.073	60	−0.023	0.869	55	**0.317**	**0.015**	**59**			
CPOST	0.110	0.405	60	0.027	0.840	59	0.039	0.776	56	0.118	0.378	58			
SCC	**0.290**	**0.024**	**60**	0.168	0.200	60	−0.041	0.759	57	0.085	0.517	60			

*Note*: Pearson’s correlations of metabolite concentrations, quantitative MRI parameters and spectral linewidth

*R* is the Pearson’s correlation coefficient. *N* represents number of sampled subjects. *p* represents the *p*-value

Bold indicates *p* < 0.05. Region of interest (*ROI*): hand motor cortical area (*HCA*), postcentral gyrus (*PCEN*), posterior cingulate gyrus (*CING*), splenium of the corpus callosum (*SCC*), thalamus (*THAL*), occipital area (*OC*), lateral temporal lobe (*TLAT*), medial temporal lobe (*TMED*), insular gyrus (*INS*), hippocampus (*HIP*), cerebellar anterior lobe (*CANT*) and cerebellar posterior lobe (*CPOST*)

**Table 2:** Results of linear regression analysis for cases with significant correlations between measured parameters and age

ROI	Parameter	*R*	*p*	*N*	Intercept	Slope	Decade variation (%)
TLAT	*NAA*	−0.355	0.0063	58	11.91	−0.038	−3.43
OC	*NAA*	−0.355	0.0054	60	12.19	−0.033	−2.86
CING	*NAA*	−0.474	0.0001	60	10.39	−0.032	−3.26
TMED	*NAA*	−0.485	0.0002	53	9.77	−0.043	−4.83
HIP	*NAA*	−0.370	0.0039	59	8.96	−0.027	−3.21
THAL	*NAA*	−0.432	0.0006	60	8.86	−0.029	−3.50
INS	*NAA*	−0.494	0.0001	60	12.51	−0.049	−4.25
CPOST	*NAA*	−0.268	0.0459	56	10.85	−0.022	−2.11
THAL	*tCho*	0.255	0.0494	60	1.51	0.006	3.69
SCC	*tCho*	0.382	0.0033	57	1.12	0.009	6.90
HCA	*tCr*	0.472	0.0002	59	6.17	0.042	5.99
OC	*tCr*	0.390	0.0020	60	7.05	0.034	4.42
HIP	*tCr*	0.369	0.0041	59	6.98	0.027	3.59
SCC	*tCr*	0.368	0.0045	58	4.18	0.037	7.52
TMED	*Glx*	−0.317	0.0266	49	8.69	−0.035	−4.38
SCC	*Glx*	0.319	0.0188	54	5.12	0.043	7.20
HCA	*mI*	0.376	0.0071	50	3.39	0.036	8.81
THAL	*mI*	0.302	0.0250	55	3.53	0.027	6.64
SCC	*mI*	0.536	0.0000	57	2.09	0.056	17.00
TLAT	*sLW*	0.285	0.0300	58	8.31	0.022	2.46
CING	*sLW*	0.509	0.0000	59	5.79	0.024	3.83
TMED	*sLW*	0.408	0.0024	53	7.24	0.031	3.95
HIP	*sLW*	0.276	0.0343	59	7.86	0.024	2.88
THAL	*sLW*	0.273	0.0348	60	7.73	0.018	2.28
CANT	*sLW*	0.316	0.0147	59	6.26	0.030	4.34
HCA	*T1*	0.353	0.0057	60	788.91	2.687	3.19
PCEN	*T1*	0.372	0.0044	57	814.30	2.438	2.83
CING	*T1*	0.317	0.0207	53	1226.57	5.873	4.37
SCC	*T1*	0.290	0.0244	60	1192.60	2.229	1.80
HCA	*T2*	−0.309	0.0183	58	125.34	−0.120	−0.97
HCA	*T2’*	−0.407	0.0023	54	73.27	−0.249	−3.65
CING	*T2’*	−0.370	0.0046	57	84.24	−0.269	−3.41

*Note*: Linear regression analysis for significant correlations between measured parameters and age

*R* is the Pearson’s correlation coefficient. *N* represents number of sampled subjects. *p* represents the *p*-value. Region of interest (ROI): hand motor cortical area (*HCA*), postcentral gyrus (*PCEN*), posterior cingulate gyrus (*CING*), splenium of the corpus callosum (*SCC*), thalamus (*THAL*), occipital area (*OC*), lateral temporal lobe (*TLAT*), medial temporal lobe (*TMED*), insular gyrus (*INS*), hippocampus (*HIP*), cerebellar anterior lobe (*CANT*) and cerebellar posterior lobe (*CPOST*)

While Supplemental Table 1 provides a rough impression about the regional variations of the measured parameters, when age varied from the third age decade to the seventh age decade, the results presented in Table 1 and 2, and Fig. 2a–c revealed changes of the measured values associated with increasing age. The [NAA] decreased significantly in eight of twelve brain regions (TLAT, OC, CING, TMED, HIP, THAL, INS, CPOST, *p* = 0.006–0.046), with a varying rate ranging from −2.11% to −4.83% per decade (p. d.). The [tCho] showed a significant increase in SCC (6.9% p. d., *p* = 0.003) and in THAL (3.69% p. d., *p* = 0.049); The [tCr] showed a significant increase in four brain regions (HCA, OC, HIP, and SCC, *p* = 0.0002–0.005), with a varying rate ranging from 3.59% to 7.52% p. d. The [Glx] showed a significant decrease in TMED (−4.38% p. d., *p* = 0.027) and an increase in SCC (7.20% p. d., *p* = 0.019). The [mI] demonstrated a significant increase in three brain regions (HCA, THAL, SCC, *p* = 0.00002–0.025) with a varying rate ranging from 6.64% to 17% p. d. Concomitant with the changes in metabolite concentrations, the spectral linewidth revealed significant age-dependent increases in six ROIs (TLAT, CING, TMED, HIP, THAL and CANT, *p* = 0.00004–0.035). Similarly, the qMRI parameters revealed regional dependent inhomogeneous variations associated with increased age in several brain areas: T1 increased significantly in four ROIs (HCA, PCEN, CING and SCC, *p* = 0.004–0.024) with a ratio ranging from 1.80% to 4.37% p. d.; T2 decreased in one ROI (HCA, *p* = 0.018) with a ratio of −0.97% p. d. and T2’ decreased significantly in two ROIs, i.e. in HCA (−3.65% p. d., *p* = 0.002) and in CING (−3.41% p. d., *p* = 0.005).

As shown in Table 2, three of twelve ROIs (HCA, CING, and SCC) revealed age-related changes both in metabolic parameters and in tissue relaxation times. The corresponding Pearsonʼs correlation test revealed a negative correlation between tCr concentrations and T2’ relaxation time (R = −0.28, *p* = 0.043) in HCA, and positive correlations of T1 relaxation time to Cho (R = 0.29, *p* = 0.029), to tCr (R = 0.34, *p* = 0.009), to mI (R = 0.47, *p* = 0.0002), and to Glx (R = 0.29, *p* = 0.034) in SCC.

## Discussion

In this study, by using combined short echo wbMRSI and qMRI methods, brain regional metabolites and longitudinal and transverse relaxation times of brain tissue have been simultaneously determined from multiple structures of healthy aging human brain. The concentrations of the metabolites NAA, Cho, tCr, Glx, and mI as well as the T2, and T2’ values found in this study are in line with those previously published by studies using only qMRI measurements [2, 21], wbMRSI [10, 16, 22] or only single voxel spectroscopy (SVS) [23, 24]. Previously published results for the association of T1 values with age were heterogeneous [25] and showed some discrepancy to the present results. The reason for this heterogeneity may be the differences in methods for acquiring a T1 map [26] and differences in age and number of subjects.

The major findings of this study are the simultaneously observed regional inhomogeneous changes of metabolite concentrations and tissue relaxation times in the aging human brain. As shown in Table 2, brain metabolite concentrations changed with age within the observed five decades of age, although with variable rates depending on the metabolite and selected brain region. These observations indicate that in healthy aging human brain both metabolic and microstructural alterations occur in a region-specific manner.

### Metabolic Changes

The observed decreases of [NAA] with age are consistent with those reported previously, e.g., a decrease in NAA with age variably in specific areas of the brain [10, 16, 27–31], or over different brain lobes [11, 20] and could be interpreted as age-related regional loss of neuronal density or neuronal metabolic attenuation [6], and considered as a possible reason for cognitive alterations with age [28, 32, 33]. Our observation of increasing [tCr] and [Cho] with age but in much fewer brain areas than in [NAA] have also been reported in previous studies [6, 7]. The increased [tCr] could be interpreted as possible upregulation of creatine for compensation of altered energy demand, considering tCr as a marker for energy metabolism and as an energy reservoir [34], and increased [Cho] could be interpreted as altered cell membrane turnover in the aging process [20, 35]. To date, there are relatively fewer reports on changes of [mI] and [Glx] in aging human brain than those on changes of [NAA], [Cho], and [tCr], and the reported results are often inconclusive [6]. Nevertheless, an increase of myo-inositol was also reported in several previous studies [20, 30, 36, 37]. In line with the present observed increases of [mI], with age, Yang et al. found higher [mI] in brain regions of the senior citizens in comparison to those of younger adults using MRS on brain centrum semiovale, hippocampus and thalamus [37]. Age-related increase of [mI] has been suggested as possible gliosis or increase of glial cell volume, indicating a possible link between gliosis in brain and aging [20, 30, 37]. The observed significant increase of [Glx] in TMED and decrease in SCC may indicate age-independent removal or activation of the neurotransmitter, as explained by Cleeland et al., who noted the difficulty of measuring Glx and suggested that glutamate and glutamine act as opposing neurotransmitters in processes of astrogliosis and neurodegeneration within the brain [6]. As a consequence, the measurement of Glx may not be a true representation of the neurometabolic changes happening in the brain.

### Microstructural Changes

Corresponding to the measured changes of brain tissue relaxation times shown in Table 2, alterations of T2, T2’ and T1 with age in human brain have also been reported in previous studies in several regions of the aging brain [16, 25, 38–40]. A decrease of T2’ in cortical regions has been reported and linked to increased deoxyhemoglobin in elderly subjects caused by altered cerebral autoregulation of blood circulation [38] as well as to an increased iron deposition [16, 41], while both increases and decreases of T2 have been considered to reflect an age-related change of free water under different physiological conditions [1, 16]. Accordingly, our observation of age-related T2’ and T2 decreases in HCA and T2’ decrease in CING may be interpreted in a similar way. The changes of the T1 relaxation time have been related to multiple factors, such as altered water content, iron deposition, and myelination in brain parenchyma [25, 42, 43]. The increases of T1 relaxation time found in our study may be explained due to altered myelination and axonal loss in white matter during aging as a natural process [25, 40], considering the fact that we only saw trends in changes in cortical and subcortical tissue (HMA, PCEN, CING) and in pure white matter (SCC). In addition to an increase of T1 during natural aging, focal changes in T1 relaxation times have also been found in neurodegenerative diseases such as Parkinsonʼs disease [44] and Alzheimerʼs disease [45], therefore, establishing normative values for T1 measurements is helpful for research on neurodegenerative diseases.

### Combined Changes

Interestingly, this study identified several brain areas that had both significant age-related changes of metabolite concentrations and brain water relaxation times, indicating a connection between metabolic and microstructural alterations in aging human brain. In the hand motor area, the observed decrease of T2’, increase of [tCR], and significant negative correlation between [tCr] and T2’, may suggest that aging in this area is associated with an increased deoxyhemoglobin (indicated by reduced T2’) and with an upregulation of creatine for compensation in altered energy demand (implied by increased [tCr]). These metabolic and microstructural changes may explain the decrease of motor function with age. The observed positive correlations of T1 relaxation time to [Cho], [tCr], [Glx], and [mI] in SCC may indicate that normal aging is associated with altered myelination and axonal loss in white matter (indicated by altered T1) with associated distinctive metabolic alterations related to membrane turnover (increased [Cho]), energy metabolism (increased [tCr]), neurotransmitter function (increased [Glx]), and gliosis (increased [mI]) in the splenium of the corpus callosum. Considering that the corpus callosum has been shown to be involved in visuospatial information transfer, language, reading and calculation scores, intelligence quotient, behavior and consciousness [46], the observed correlated metabolic and microstructural alterations may be the reason behind physiological decline in these domains of life with aging.

To our knowledge, only one study to date has examined possible associations of the metabolite changes with alterations of tissue relaxation times in the aging human brain, in which brain T2 and T2’ relaxation time was measured along with only three brain metabolites (NAA, Cho, and tCr) [16]. The results of this study show similar findings of decreased T2’ relaxation time in HCA and decreased [NAA] in OCC, although our finding for T2’ in SCC contradicts the data of Eylers et al., as the SCC regions of interest in our study are localized more laterally than in the previous publication.

## Limitation and Conclusion

This study is limited by the number of subjects (*N* = 60) as well as the wide age range (21–69 years), although the selection was sex-balanced with an evenly distributed age. Further studies with a larger sample size and broader age range will be necessary to validate the observed influences of age on metabolic and microstructural changes, and to build up a reliable reference database.

In conclusion, this study has shown that that there are both metabolic and microstructural changes in the normal aging human brain, which are regionally variant. These combined aging effects may reflect age-related decline in different cognitive and motor domains of life in the elderly population.

### Supplementary Information

The supplementary information provides an extensive depiction of the regional distribution of parameters across the analyzed age groups, using scatter plots and a tabular format.
