# Supplementary material for: Microstructural and Metabolic Changes in Normal Aging Human Brain Studied with Combined Whole-Brain MR Spectroscopic Imaging and Quantitative MR Imaging
Source: Clin Neuroradiol. 2023 Jun 19;33(4):993–1005. doi: 10.1007/s00062-023-01300-3 (PMC10654209; doi:10.1007/s00062-023-01300-3)
Supplement: Supplementary file 1 — The supplementary information provides an extensive depiction of the regional distribution of parameters across the analyzed age groups, using scatter plots and a tabular format. [file 62_2023_1300_MOESM1_ESM.docx]

**
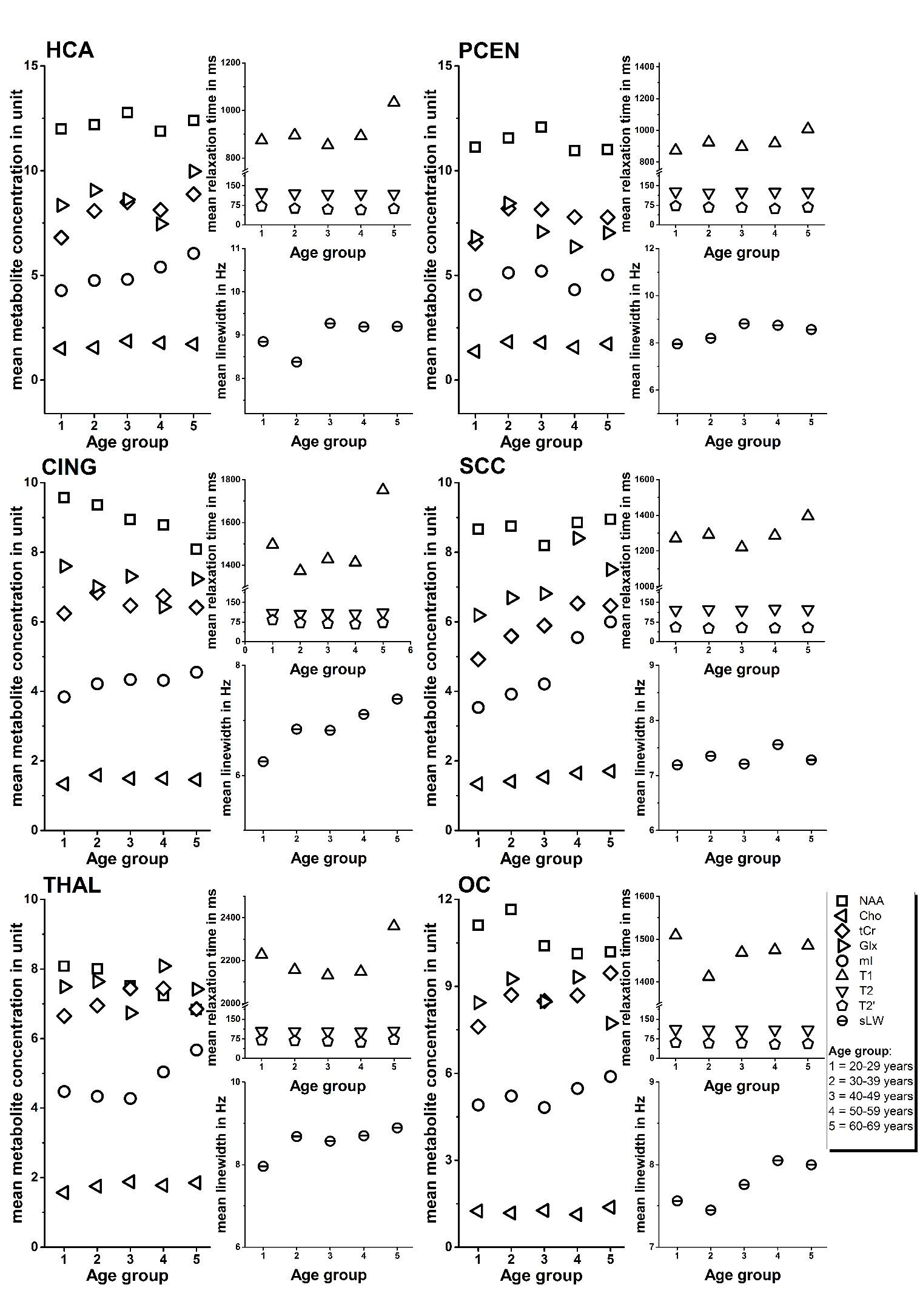
Supplement:**

**Supplement figure 1.** Regional parameters of agegroups in HCA, PCEN, CING, SCC,

THAL and OC as scatterplots


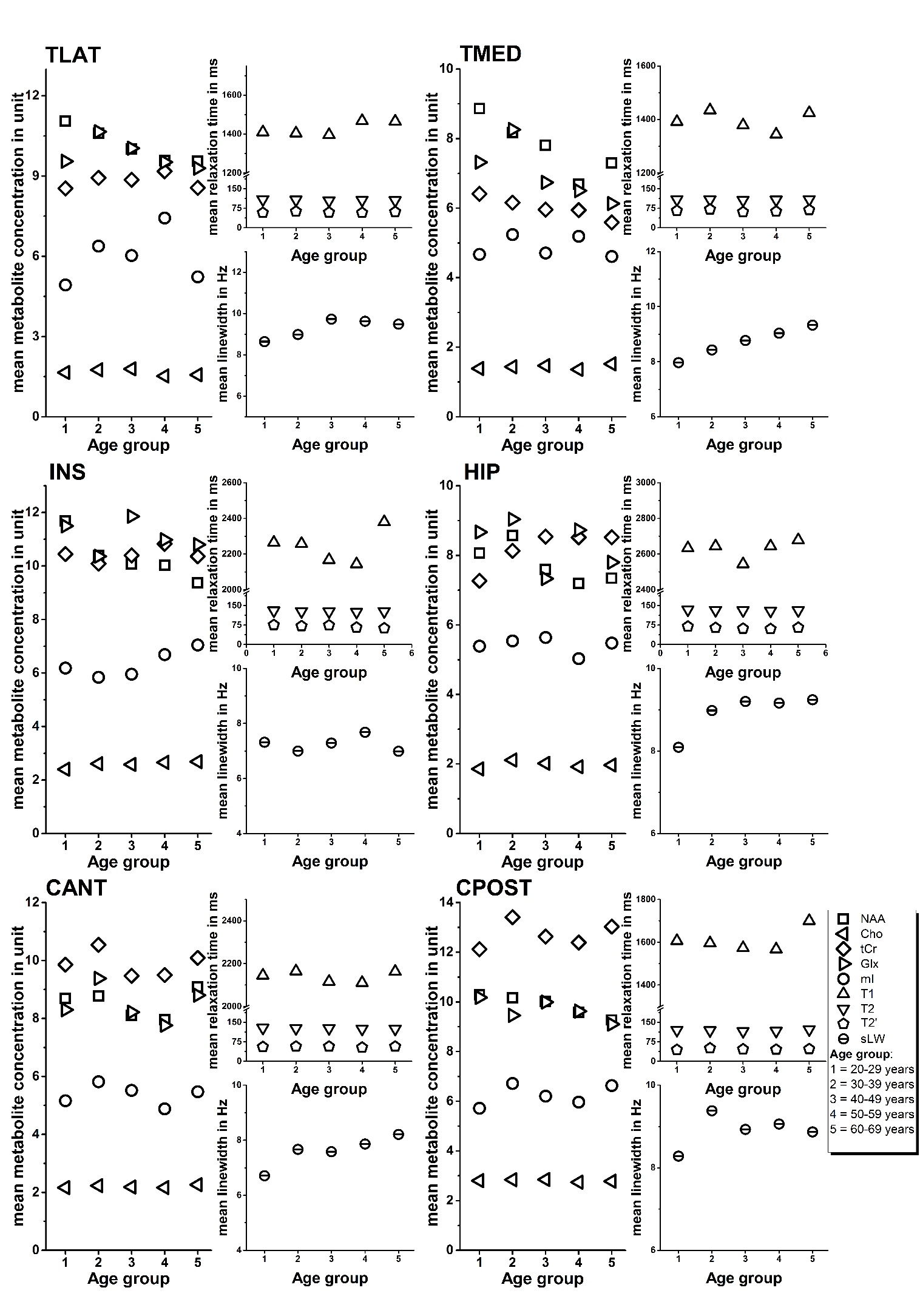


**Supplement figure 2.** Regional parameters of agegroups in TLAT, TMED, INS, HIP, CANT

and CPOST as scatterplots

**Supplement Table 1. Regional parameters in agegroups in all ROI**

|  |  |  | **NAA** |  |  | **Cho** |  |  | **tCr** |  |  | **Glx** |  |  | **mI** |  |  | **sLW** |  |  | **T1** |  |  | **T2** |  |  | **T2‘** |  |
| --- | --- | --- | --- | --- | --- | --- | --- | --- | --- | --- | --- | --- | --- | --- | --- | --- | --- | --- | --- | --- | --- | --- | --- | --- | --- | --- | --- | --- |
|  |  | N | Mean | SD | N | Mean | SD | N | Mean | SD | N | Mean | SD | N | Mean | SD | N | Mean | SD | N | Mean | SD | N | Mean | SD | N | Mean | SD |
| HCA | 20-29 | 12 | 12.00 | 1.12 | 12 | 1.51 | 0.42 | 12 | 6.80 | 1.48 | 11 | 8.35 | 1.90 | 10 | 4.28 | 1.28 | 12 | 8.85 | 1.25 | 12 | 875.02 | 95.00 | 9 | 125.36 | 4.05 | 12 | 71.10 | 8.62 |
|  | 30-39 | 12 | 12.19 | 1.15 | 12 | 1.55 | 0.36 | 12 | 8.08 | 1.25 | 11 | 9.06 | 2.05 | 10 | 4.76 | 1.02 | 12 | 8.38 | 1.17 | 12 | 895.95 | 74.08 | 12 | 119.90 | 9.35 | 11 | 62.05 | 7.63 |
|  | 40-49 | 11 | 12.77 | 1.01 | 11 | 1.87 | 0.48 | 11 | 8.49 | 0.90 | 11 | 8.62 | 2.13 | 11 | 4.81 | 1.19 | 11 | 9.27 | 0.70 | 12 | 854.21 | 64.71 | 12 | 118.22 | 3.30 | 11 | 58.54 | 9.02 |
|  | 50-59 | 12 | 11.89 | 1.62 | 12 | 1.78 | 0.50 | 12 | 8.13 | 0.80 | 11 | 7.46 | 3.00 | 10 | 5.40 | 1.78 | 12 | 9.19 | 1.64 | 12 | 893.02 | 114.31 | 11 | 118.81 | 4.35 | 11 | 57.26 | 6.69 |
|  | 60-69 | 12 | 12.40 | 1.38 | 12 | 1.72 | 0.32 | 12 | 8.88 | 1.42 | 11 | 9.97 | 2.74 | 9 | 6.05 | 1.68 | 12 | 9.20 | 0.81 | 12 | 1033.62 | 139.04 | 11 | 118.70 | 5.04 | 9 | 61.51 | 8.78 |
| PCEN | 20-29 | 12 | 11.12 | 1.25 | 12 | 1.37 | 0.26 | 12 | 6.53 | 0.89 | 12 | 6.83 | 2.03 | 11 | 4.07 | 1.08 | 12 | 7.96 | 0.82 | 12 | 873.91 | 96.00 | 9 | 127.83 | 5.35 | 11 | 71.53 | 10.03 |
|  | 30-39 | 12 | 11.57 | 1.58 | 12 | 1.83 | 0.38 | 12 | 8.19 | 1.23 | 12 | 8.45 | 2.93 | 10 | 5.12 | 1.10 | 12 | 8.20 | 1.19 | 12 | 923.93 | 93.73 | 12 | 123.52 | 6.67 | 11 | 66.67 | 9.60 |
|  | 40-49 | 12 | 12.07 | 1.20 | 12 | 1.79 | 0.33 | 12 | 8.15 | 1.59 | 11 | 7.09 | 2.10 | 12 | 5.21 | 1.06 | 12 | 8.81 | 1.37 | 12 | 895.19 | 47.17 | 12 | 126.64 | 7.13 | 12 | 65.35 | 9.50 |
|  | 50-59 | 12 | 10.96 | 1.48 | 12 | 1.57 | 0.33 | 12 | 7.79 | 0.77 | 11 | 6.36 | 2.16 | 12 | 4.32 | 0.96 | 12 | 8.75 | 1.24 | 10 | 918.13 | 122.50 | 11 | 126.09 | 6.29 | 9 | 61.72 | 5.24 |
|  | 60-69 | 12 | 11.02 | 1.84 | 12 | 1.73 | 0.40 | 12 | 7.78 | 1.27 | 12 | 7.03 | 1.25 | 12 | 5.02 | 1.71 | 12 | 8.57 | 1.50 | 11 | 1008.07 | 93.93 | 12 | 126.45 | 6.39 | 10 | 66.74 | 7.23 |
| TLAT | 20-29 | 12 | 11.06 | 1.43 | 12 | 1.65 | 0.40 | 12 | 8.54 | 1.67 | 12 | 9.55 | 1.57 | 12 | 4.93 | 1.11 | 12 | 8.64 | 1.19 | 12 | 1410.28 | 162.91 | 9 | 109.81 | 4.67 | 11 | 57.30 | 5.86 |
|  | 30-39 | 12 | 10.61 | 1.15 | 11 | 1.75 | 0.45 | 12 | 8.93 | 0.63 | 12 | 10.66 | 3.28 | 11 | 6.38 | 1.52 | 12 | 8.99 | 0.92 | 12 | 1403.83 | 118.69 | 12 | 107.62 | 4.90 | 10 | 62.67 | 8.87 |
|  | 40-49 | 12 | 10.00 | 1.60 | 11 | 1.79 | 0.33 | 12 | 8.86 | 1.34 | 12 | 10.04 | 2.75 | 11 | 6.03 | 1.80 | 12 | 9.73 | 0.79 | 12 | 1396.87 | 166.32 | 12 | 105.01 | 5.14 | 12 | 59.15 | 15.34 |
|  | 50-59 | 11 | 9.57 | 1.55 | 10 | 1.52 | 0.44 | 11 | 9.18 | 1.60 | 11 | 9.52 | 2.74 | 10 | 7.43 | 2.18 | 11 | 9.63 | 1.16 | 12 | 1468.62 | 224.84 | 12 | 107.23 | 4.14 | 11 | 57.69 | 9.79 |
|  | 60-69 | 11 | 9.56 | 2.15 | 11 | 1.57 | 0.44 | 11 | 8.56 | 1.59 | 11 | 9.29 | 1.84 | 11 | 5.23 | 1.96 | 11 | 9.49 | 1.45 | 12 | 1465.27 | 142.99 | 12 | 105.80 | 4.76 | 9 | 61.58 | 17.62 |
| OC | 20-29 | 12 | 11.11 | 0.89 | 12 | 1.25 | 0.42 | 12 | 7.61 | 0.82 | 12 | 8.44 | 1.33 | 12 | 4.91 | 2.03 | 12 | 7.56 | 1.87 | 12 | 1509.39 | 195.31 | 6 | 112.19 | 5.66 | 12 | 59.39 | 8.21 |
|  | 30-39 | 12 | 11.65 | 1.26 | 12 | 1.18 | 0.31 | 12 | 8.70 | 1.08 | 12 | 9.26 | 2.06 | 12 | 5.22 | 0.84 | 12 | 7.45 | 1.41 | 12 | 1412.29 | 247.98 | 10 | 110.10 | 7.43 | 12 | 56.74 | 7.11 |
|  | 40-49 | 12 | 10.40 | 0.81 | 12 | 1.27 | 0.32 | 12 | 8.50 | 0.91 | 12 | 8.47 | 1.38 | 12 | 4.82 | 1.08 | 12 | 7.76 | 0.88 | 12 | 1468.79 | 123.68 | 10 | 109.22 | 6.45 | 11 | 57.30 | 11.92 |
|  | 50-59 | 12 | 10.12 | 2.12 | 11 | 1.13 | 0.18 | 12 | 8.69 | 1.42 | 12 | 9.32 | 2.72 | 12 | 5.48 | 1.19 | 12 | 8.05 | 1.47 | 11 | 1474.59 | 213.71 | 8 | 109.70 | 10.44 | 11 | 53.13 | 6.36 |
|  | 60-69 | 12 | 10.19 | 1.20 | 12 | 1.38 | 0.48 | 12 | 9.46 | 1.75 | 11 | 7.73 | 1.34 | 12 | 5.89 | 1.69 | 12 | 8.00 | 0.96 | 11 | 1485.35 | 156.64 | 11 | 109.25 | 6.07 | 10 | 55.70 | 7.84 |
| CING | 20-29 | 12 | 9.57 | 0.72 | 12 | 1.34 | 0.20 | 12 | 6.24 | 0.76 | 12 | 7.60 | 1.33 | 11 | 3.84 | 0.92 | 12 | 6.25 | 0.52 | 12 | 1496.86 | 320.31 | 9 | 110.27 | 3.64 | 12 | 82.22 | 10.33 |
|  | 30-39 | 12 | 9.36 | 0.83 | 12 | 1.59 | 0.37 | 12 | 6.84 | 0.74 | 12 | 7.01 | 1.22 | 10 | 4.22 | 0.63 | 12 | 6.84 | 0.79 | 11 | 1373.45 | 228.33 | 12 | 105.72 | 5.51 | 11 | 71.05 | 8.07 |
|  | 40-49 | 12 | 8.94 | 0.65 | 12 | 1.49 | 0.12 | 12 | 6.47 | 0.97 | 12 | 7.31 | 1.51 | 12 | 4.34 | 0.94 | 12 | 6.82 | 0.63 | 10 | 1429.44 | 262.67 | 12 | 108.47 | 4.51 | 12 | 69.11 | 12.20 |
|  | 50-59 | 12 | 8.79 | 1.09 | 12 | 1.50 | 0.25 | 12 | 6.74 | 1.06 | 10 | 6.43 | 1.28 | 11 | 4.32 | 1.11 | 11 | 7.11 | 0.53 | 11 | 1413.16 | 202.35 | 12 | 106.54 | 4.85 | 11 | 66.19 | 7.32 |
|  | 60-69 | 12 | 8.08 | 1.19 | 12 | 1.46 | 0.20 | 12 | 6.41 | 0.68 | 12 | 7.23 | 1.05 | 11 | 4.55 | 0.92 | 12 | 7.39 | 0.66 | 12 | 1751.16 | 249.73 | 12 | 110.81 | 7.64 | 11 | 71.78 | 11.36 |
| TMED | 20-29 | 12 | 8.86 | 0.95 | 12 | 1.39 | 0.21 | 12 | 6.41 | 0.58 | 12 | 7.32 | 1.17 | 12 | 4.67 | 1.68 | 12 | 7.97 | 1.23 | 12 | 1391.94 | 125.74 | 9 | 109.79 | 3.76 | 12 | 65.75 | 7.89 |
|  | 30-39 | 11 | 8.17 | 1.48 | 11 | 1.44 | 0.30 | 11 | 6.16 | 1.39 | 11 | 8.26 | 1.73 | 11 | 5.24 | 1.13 | 11 | 8.43 | 0.96 | 11 | 1434.47 | 155.51 | 12 | 108.68 | 5.00 | 7 | 71.23 | 7.59 |
|  | 40-49 | 11 | 7.81 | 1.02 | 11 | 1.47 | 0.28 | 11 | 5.96 | 1.10 | 11 | 6.74 | 1.80 | 11 | 4.71 | 1.11 | 11 | 8.77 | 1.21 | 12 | 1379.00 | 104.02 | 12 | 107.43 | 4.31 | 9 | 60.74 | 8.51 |
|  | 50-59 | 11 | 6.69 | 1.10 | 10 | 1.36 | 0.35 | 10 | 5.94 | 1.12 | 8 | 6.50 | 1.41 | 9 | 5.19 | 1.46 | 11 | 9.04 | 1.08 | 12 | 1344.18 | 60.38 | 12 | 107.94 | 4.59 | 9 | 62.63 | 11.21 |
|  | 60-69 | 8 | 7.30 | 1.31 | 7 | 1.52 | 0.17 | 8 | 5.59 | 1.18 | 7 | 6.13 | 1.94 | 8 | 4.61 | 1.04 | 8 | 9.33 | 1.09 | 12 | 1424.80 | 98.09 | 12 | 109.56 | 5.18 | 9 | 69.02 | 8.98 |
| HIP | 20-29 | 12 | 8.06 | 0.76 | 12 | 1.86 | 0.32 | 12 | 7.27 | 0.59 | 12 | 8.67 | 1.45 | 12 | 5.39 | 1.30 | 12 | 8.09 | 1.41 | 12 | 2634.16 | 264.50 | 9 | 135.53 | 7.37 | 12 | 69.74 | 6.08 |
|  | 30-39 | 11 | 8.57 | 0.64 | 11 | 2.11 | 0.25 | 11 | 8.13 | 1.00 | 11 | 9.04 | 2.39 | 11 | 5.54 | 1.17 | 11 | 8.98 | 1.25 | 11 | 2644.86 | 212.14 | 12 | 132.03 | 7.36 | 12 | 64.46 | 12.34 |
|  | 40-49 | 12 | 7.59 | 1.22 | 12 | 2.02 | 0.33 | 12 | 8.54 | 1.01 | 10 | 7.33 | 1.62 | 12 | 5.64 | 1.49 | 12 | 9.20 | 1.13 | 12 | 2544.07 | 132.66 | 12 | 131.67 | 7.68 | 12 | 61.32 | 8.96 |
|  | 50-59 | 12 | 7.19 | 0.96 | 12 | 1.92 | 0.51 | 12 | 8.52 | 1.26 | 11 | 8.73 | 1.89 | 11 | 5.03 | 1.52 | 12 | 9.16 | 1.42 | 12 | 2644.17 | 284.62 | 12 | 130.39 | 5.65 | 10 | 59.64 | 12.77 |
|  | 60-69 | 12 | 7.34 | 1.36 | 12 | 1.97 | 0.35 | 12 | 8.52 | 1.20 | 12 | 7.80 | 2.36 | 12 | 5.48 | 1.24 | 12 | 9.24 | 1.22 | 12 | 2678.81 | 240.73 | 12 | 131.89 | 7.08 | 11 | 65.39 | 14.54 |
| THAL | 20-29 | 12 | 8.08 | 1.04 | 12 | 1.57 | 0.30 | 12 | 6.65 | 0.89 | 12 | 7.49 | 1.67 | 10 | 4.48 | 1.16 | 12 | 7.96 | 1.04 | 12 | 2227.15 | 273.76 | 9 | 103.88 | 4.07 | 12 | 68.83 | 5.51 |
|  | 30-39 | 12 | 8.01 | 1.00 | 12 | 1.75 | 0.28 | 12 | 6.95 | 0.89 | 12 | 7.64 | 1.44 | 12 | 4.34 | 1.11 | 12 | 8.68 | 0.97 | 12 | 2156.97 | 283.95 | 12 | 102.81 | 4.58 | 11 | 66.90 | 15.25 |
|  | 40-49 | 12 | 7.51 | 0.64 | 12 | 1.88 | 0.39 | 12 | 7.44 | 0.96 | 12 | 6.74 | 1.83 | 11 | 4.28 | 0.82 | 12 | 8.57 | 0.78 | 12 | 2132.18 | 114.26 | 12 | 102.85 | 4.46 | 12 | 64.91 | 9.81 |
|  | 50-59 | 12 | 7.23 | 1.24 | 12 | 1.78 | 0.27 | 12 | 7.44 | 1.22 | 12 | 8.09 | 2.57 | 11 | 5.04 | 1.64 | 12 | 8.70 | 1.09 | 12 | 2148.16 | 218.51 | 12 | 101.85 | 5.81 | 11 | 60.60 | 10.56 |
|  | 60-69 | 12 | 6.83 | 0.76 | 12 | 1.85 | 0.40 | 12 | 6.85 | 1.12 | 11 | 7.42 | 1.95 | 11 | 5.67 | 1.70 | 12 | 8.89 | 1.18 | 12 | 2360.71 | 257.65 | 12 | 106.03 | 5.99 | 11 | 70.86 | 12.80 |
| INS | 20-29 | 12 | 11.69 | 1.39 | 12 | 2.40 | 0.43 | 12 | 10.45 | 1.27 | 12 | 11.50 | 1.91 | 12 | 6.19 | 1.83 | 12 | 7.32 | 1.29 | 12 | 2264.38 | 174.57 | 9 | 131.95 | 8.82 | 11 | 75.08 | 11.47 |
|  | 30-39 | 12 | 10.39 | 1.43 | 12 | 2.61 | 0.40 | 12 | 10.09 | 0.84 | 12 | 10.36 | 1.97 | 12 | 5.84 | 1.25 | 12 | 7.00 | 0.94 | 11 | 2258.23 | 119.08 | 12 | 128.10 | 5.86 | 10 | 70.65 | 16.37 |
|  | 40-49 | 12 | 10.07 | 1.19 | 12 | 2.58 | 0.57 | 12 | 10.40 | 1.79 | 12 | 11.86 | 3.37 | 12 | 5.96 | 1.46 | 12 | 7.29 | 0.71 | 12 | 2167.81 | 108.15 | 12 | 127.22 | 9.69 | 9 | 73.80 | 10.52 |
|  | 50-59 | 12 | 10.03 | 1.52 | 12 | 2.66 | 0.53 | 12 | 10.83 | 2.03 | 11 | 10.98 | 1.74 | 10 | 6.69 | 1.51 | 12 | 7.68 | 0.88 | 12 | 2143.87 | 114.26 | 9 | 125.89 | 7.78 | 9 | 65.66 | 9.41 |
|  | 60-69 | 12 | 9.38 | 1.13 | 12 | 2.69 | 0.41 | 12 | 10.36 | 1.57 | 12 | 10.80 | 2.93 | 12 | 7.05 | 1.72 | 12 | 6.99 | 0.50 | 12 | 2380.13 | 220.46 | 8 | 127.90 | 8.75 | 5 | 62.98 | 15.58 |
| CANT | 20-29 | 11 | 8.69 | 0.62 | 11 | 2.16 | 0.18 | 11 | 9.86 | 0.77 | 10 | 8.30 | 1.78 | 10 | 5.16 | 0.68 | 11 | 6.71 | 0.76 | 12 | 2144.92 | 176.77 | 9 | 129.89 | 5.72 | 12 | 55.54 | 9.23 |
|  | 30-39 | 12 | 8.77 | 0.86 | 12 | 2.23 | 0.30 | 12 | 10.54 | 0.78 | 10 | 9.38 | 1.45 | 11 | 5.82 | 1.09 | 12 | 7.67 | 1.30 | 11 | 2164.74 | 142.08 | 12 | 127.41 | 6.10 | 11 | 56.56 | 11.48 |
|  | 40-49 | 12 | 8.10 | 1.18 | 12 | 2.18 | 0.25 | 12 | 9.47 | 0.56 | 11 | 8.22 | 1.31 | 12 | 5.52 | 0.91 | 12 | 7.58 | 1.70 | 12 | 2116.51 | 154.99 | 12 | 126.99 | 5.70 | 11 | 56.98 | 12.49 |
|  | 50-59 | 12 | 7.96 | 1.46 | 12 | 2.16 | 0.28 | 12 | 9.50 | 1.30 | 10 | 7.76 | 1.82 | 11 | 4.88 | 0.94 | 12 | 7.86 | 1.37 | 12 | 2111.63 | 158.96 | 12 | 125.22 | 4.43 | 11 | 53.53 | 10.23 |
|  | 60-69 | 12 | 9.08 | 0.64 | 12 | 2.26 | 0.26 | 12 | 10.09 | 1.18 | 11 | 8.80 | 1.37 | 12 | 5.47 | 0.98 | 12 | 8.21 | 1.65 | 12 | 2162.92 | 185.98 | 12 | 125.22 | 5.73 | 10 | 56.75 | 9.27 |
| CPOST | 20-29 | 11 | 10.29 | 1.05 | 11 | 2.80 | 0.20 | 11 | 12.12 | 1.39 | 11 | 10.18 | 1.59 | 11 | 5.72 | 1.17 | 11 | 8.28 | 1.58 | 12 | 1606.84 | 246.54 | 9 | 122.43 | 6.12 | 12 | 44.23 | 9.97 |
|  | 30-39 | 12 | 10.16 | 1.36 | 12 | 2.84 | 0.45 | 12 | 13.41 | 1.64 | 12 | 9.46 | 1.52 | 12 | 6.72 | 1.28 | 12 | 9.38 | 1.18 | 12 | 1595.84 | 85.57 | 11 | 119.18 | 5.63 | 11 | 50.12 | 8.45 |
|  | 40-49 | 12 | 10.02 | 0.73 | 12 | 2.85 | 0.33 | 12 | 12.63 | 1.28 | 11 | 9.99 | 1.24 | 12 | 6.21 | 1.41 | 12 | 8.93 | 1.29 | 12 | 1573.38 | 140.30 | 12 | 116.27 | 2.91 | 12 | 46.83 | 11.35 |
|  | 50-59 | 10 | 9.57 | 1.15 | 11 | 2.74 | 0.36 | 11 | 12.39 | 2.48 | 9 | 9.63 | 1.96 | 10 | 5.97 | 1.95 | 11 | 9.06 | 1.13 | 12 | 1567.38 | 248.71 | 12 | 116.67 | 3.87 | 11 | 45.17 | 8.21 |
|  | 60-69 | 11 | 9.27 | 1.61 | 12 | 2.78 | 0.47 | 12 | 13.03 | 1.45 | 9 | 9.10 | 1.48 | 12 | 6.63 | 1.36 | 12 | 8.87 | 1.47 | 12 | 1700.43 | 108.02 | 12 | 122.54 | 5.78 | 10 | 47.17 | 7.72 |
| SCC | 20-29 | 12 | 8.67 | 1.21 | 12 | 1.34 | 0.29 | 12 | 4.92 | 0.79 | 12 | 6.19 | 1.24 | 11 | 3.54 | 0.52 | 12 | 7.19 | 0.62 | 12 | 1270.99 | 121.93 | 9 | 121.09 | 9.21 | 12 | 53.95 | 4.38 |
|  | 30-39 | 12 | 8.75 | 1.15 | 12 | 1.41 | 0.24 | 12 | 5.59 | 0.93 | 12 | 6.69 | 1.73 | 12 | 3.92 | 1.54 | 12 | 7.35 | 0.44 | 12 | 1291.48 | 95.90 | 12 | 123.15 | 11.42 | 12 | 50.61 | 8.72 |
|  | 40-49 | 12 | 8.20 | 1.40 | 12 | 1.53 | 0.40 | 12 | 5.89 | 1.09 | 11 | 6.81 | 1.65 | 12 | 4.21 | 1.14 | 12 | 7.21 | 0.63 | 12 | 1220.68 | 46.24 | 12 | 121.94 | 9.75 | 12 | 52.56 | 7.16 |
|  | 50-59 | 12 | 8.86 | 1.36 | 12 | 1.65 | 0.30 | 12 | 6.53 | 1.95 | 11 | 8.40 | 2.85 | 12 | 5.55 | 1.44 | 12 | 7.56 | 0.66 | 12 | 1287.99 | 119.26 | 12 | 124.91 | 8.27 | 10 | 51.01 | 5.32 |
|  | 60-69 | 10 | 8.95 | 1.86 | 9 | 1.70 | 0.43 | 10 | 6.46 | 2.22 | 8 | 7.50 | 2.33 | 10 | 6.00 | 1.54 | 12 | 7.28 | 0.60 | 12 | 1395.65 | 126.63 | 12 | 124.38 | 9.60 | 11 | 52.74 | 8.72 |

Abbreviations: N represents number of sampled subjects in each age group; SD represents the standard deviation of the mean values; hand motor cortical area (HCA), postcentral gyrus (PCEN), posterior cingulate gyrus (CING), splenium of the corpus callosum (SCC), thalamus (THAL), occipital area (OC), lateral temporal lobe (TLAT), medial temporal lobe (TMED), insular gyrus (INS), hippocampus (HIP), cerebellar anterior lobe (CANT) and cerebellar posterior lobe (CPOST)
